# Cytotoxic Drug Handling Practices Among Pharmacy Technicians in Portugal: The Dig Deeper Study

**DOI:** 10.3390/healthcare14070963

**Published:** 2026-04-06

**Authors:** Ana Reis, Vítor Silva, João José Joaquim, Cristiano Matos, Carolina Valeiro, Cristiana Freitas, Olívia R. Pereira, Ramona Mateos-Campos, Fernando Moreira

**Affiliations:** 1 REQUIMTE/LAQV, Escola Superior de Saúde, Instituto Politécnico do Porto, Rua Dr. António Bernardino de Almeida, 4200-072 Porto, Portugal; ritareis3@gmail.com; 2 Facultad de Farmacia, Universidad de Salamanca, Campus Miguel de Unamuno, 37007 Salamanca, Spain; 3 Unidade Local de Saúde (ULS) Coimbra, E.P.E., Hospitais da Universidade de Coimbra (HUC), 3004-561 Coimbra, Portugal; vahsilva@hotmail.com; 4 Associação Portuguesa de Licenciados em Farmácia (APLF), 3140-348 Coimbra, Portugal; jjj@estesc.ipc.pt (J.J.J.); cristiano.r.matos@gmail.com (C.M.); 5 Escola Superior de Tecnologia da Saúde de Coimbra, Instituto Politécnico de Coimbra, R. 5 de Outubro, 3045-043 Coimbra, Portugal; 6 European Association of Pharmacy Technicians, B-1080 Brussels, Belgium; carolinavaleiro99@gmail.com; 7 Instituto Politécnico de Bragança, Campus de Santa Apolónia, 5300-253 Bragança, Portugal; carf2013@outlook.pt; 8 Research Centre for Active Living and Wellbeing (LiveWell), Instituto Politécnico de Bragança, Campus de Santa Apolónia, 5300-253 Bragança, Portugal; oliviapereira@ipb.pt; 9 Área de Medicina Preventiva y Salud Pública, Departamento Ciencias Biomédicas y del Diagnóstico, Faculdade de Medicina, Universidad de Salamanca, 37007 Salamanca, Spain; rmateos@usal.es

**Keywords:** cytotoxic drugs, pharmacy practices, safe handling, training, occupational exposure

## Abstract

**Background**: Occupational exposure to cytotoxic drugs remains a major concern for pharmacy personnel, due to their well-established, carcinogenic, mutagenic and organ-specific effects. Despite the existence of robust international guidelines, evidence suggests substantial variability in compliance, training quality and operational conditions across healthcare settings. **Objective:** This study aimed to characterise current handling practices, assess working conditions, training, safety procedures, exposure patterns, and perceived risk factors among pharmacy technicians involved in the preparation of cytotoxic drugs in Portugal. **Methods:** A cross-sectional descriptive study was conducted using a structured questionnaire grounded in international standards (ISOPP, NIOSH, ASHP, USP <800>). The instrument was developed through literature review, expert panel validation (*n* = 42), and pre-testing. Data were collected electronically between April and May 2025 from pharmacy technicians actively handling cytotoxic drugs in Portugal. **Results:** A total of 124 valid responses were analysed. Most participants were female (78%) and under 50 years, with nearly one-third having less than one year of experience. Prolonged daily exposure (31.5% participants worked ≥ 5 h/day) extended uninterrupted handling periods (28.2% worked > 120 min), and high preparation workloads were common. While adherence to core protective measures—such as reinforced gowns, double gloves, and Class II B2 biological safety cabinets—was high, important gaps were identified, including incomplete use of closed system transfer devices, inconsistent respiratory and foot protection, limited automation, and insufficient environmental monitoring. Structured competency assessment, periodic training, and formal documentation were frequently absent. Institutional policies on reproductive risk showed strong protection for women but less clarity for male workers. **Conclusions:** Cytotoxic drug handling practices in Portugal demonstrate satisfactory adherence to fundamental protective measures but reveal significant structural and organisational gaps related to workload management, environmental monitoring, and continuous training. The absence of unified national guidance contributes to variability across institutions. These findings highlight the need for greater standardisation of occupational safety practices.

## 1. Introduction

The handling of cytotoxic drugs is an essential activity in hospital pharmacy services, however, represents one of the areas of highest occupational risk [1,2,3]. These agents have well-documented carcinogenic, mutagenic, teratogenic, and organotoxic properties [4,5,6], and can cause adverse effects even at low exposure doses, especially over prolonged periods [2,3,7,8,9]. Potential outcomes among exposed professionals include genetic alterations, reproductive dysfunctions, skin and respiratory problems, as well as persistent environmental contamination in preparation, storage, and disposal areas [8,10,11,12,13,14]. It is important to emphasize that the incidence of cancer has increased substantially over recent years, with nearly twenty million new cases diagnosed worldwide in 2022 [6].

To mitigate these occupational risks, several international organizations—such as the International Society of Oncology Pharmacy Practitioners (ISOPP) [1], the American Society of Health-System Pharmacists (ASHP) [15,16], the National Institute for Occupational Safety and Health (NIOSH) [7,17,18], USP <800> [3], European Agency for Safety & Health at Work (EU-OSHA) [19]—have published over time several documents and standards defining strict measures for both individual and collective protection. Among these, the ISOPP Standards [1], USP <800> [3], and NIOSH [7] guidelines constitute the most comprehensive and operational frameworks, as they integrate engineering controls, administrative measures, PPE, training requirements, and health surveillance criteria. These guidelines cover the mandatory use of PPE, engineering requirements, engineering controls (such as laminar flow, class II B2 cabinets or isolators, closed system transfer devices (CSTD)), limits on continuous handling time, preparation techniques, administrative controls with training standards, periodic competency assessments, and clinical criteria for the temporary suspension of activity [1,3,7]. Notably, international safety frameworks emphasize a hierarchical approach to risk mitigation, prioritizing elimination, substitution, engineering and administrative controls over reliance on personal protective equipment alone [20]. This hierarchy underpins contemporary occupational risk management strategies and provides a structured framework for evaluating safety practices in high-risk pharmaceutical settings. Despite this, evidence points to uneven and often suboptimal adherence to guidelines, influenced by structural limitations, gaps in training, the absence of harmonized institutional policies, and variability in risk perception among professionals [7,8,15,16,19,20]. The persistence of excessive workloads, prolonged handling times without adequate breaks, insufficient documentation of training, and gaps in recognizing medical conditions that increase susceptibility to exposure have already been documented [11,21,22,23,24,25,26].

In Portugal, within the European context, cytotoxic drugs are increasingly framed as carcinogenic, mutagenic and reprotoxic (CMR) agents, falling under broader occupational health and safety legislation aimed at preventing chronic exposure to hazardous medicinal products [19,27,28]. This classification highlights the importance of structured institutional measures to minimize occupational exposure. Consequently, the preparation of cytotoxic drugs has been progressively centralized and subjected to more demanding technical requirements [28,29,30]. However, empirical evidence on the actual practices of professionals, levels of training, exposure standards, and alignment with international guidelines remains limited. This lack of official data constitutes an obstacle to the implementation of fully effective and mandatory safety policies [16].

The present study aimed to provide a comprehensive characterization of the practices, working conditions, and risk perception of Pharmacy Technicians working in pharmaceutical services of Portuguese hospitals involved in handling cytotoxic drugs.

## 2. Materials and Methods

### 2.1. Study Design

A cross-sectional descriptive study was conducted to assess safety practices related to the handling of cytotoxic drugs by Pharmacy Technicians working in pharmaceutical services of Portuguese hospitals.

### 2.2. Questionnaire Development

The questionnaire development began with a comprehensive literature review focused on internationally recognised safety standards for the handling of cytotoxic drugs, including ISOPP, NIOSH, ASHP, and USP <800> [1,3,7,15,16,20]. This review aimed to ensure alignment between questionnaire items and established best practices.

Based on this framework, the main dimensions of occupational safety in cytotoxic drug handling were identified, namely, occupational health risks, engineering and administrative controls, personal protective equipment, preparation techniques, environmental contamination, and professional training.

Following the literature review, an expert consensus process was conducted using a focus group approach. A panel of forty-two experts participated in four structured rounds with clearly defined objectives.

Experts were invited by the Portuguese Association of Pharmacy Technicians (Associação Portuguesa de Licenciados em Farmácia—APLF) through email and professional social media. Eligibility criteria included holding at least a bachelor’s degree and having professional experience in hospital-based chemotherapy handling.

Based on the topics identified during the literature review and focus group sessions, a preliminary version of the questionnaire was developed, yielding an initial pool of fifty-nine questions. The preliminary questionnaire was then submitted to the experts (*n* = 42) who reviewed each item for clarity, relevance, and suitability within the hospital pharmacy context. Based on their feedback, item wording and structure were revised to eliminate ambiguous or redundant questions and to improve clarity.

Significantly, as the questionnaire was designed to capture distinct domains of occupational practice and these domains represent conceptually independent aspects rather than a single latent construct, items were analysed as independent indicators rather than components of a unified scale. Therefore, internal consistency measures were not considered appropriate. Content validity was ensured through alignment with established international guidelines, namely those from ISOPP, NIOSH, USP <800> [1,3,7,15,16,20].

### 2.3. Pre-Test and Reliability Analysis

A pre-test was conducted with the target population to assess the clarity, comprehension, and relevance of the questionnaire items, as well as its applicability in real working contexts. Feedback from this phase led to the clarification of ambiguities, reformulation of items, and optimization of response options. A total of 28 valid responses were obtained following contact through the author’s informal professional network.

### 2.4. Final Structure of the Questionnaire

The final version of the questionnaire was structured into six chapters, addressing key dimensions of safety in the handling of cytotoxic drugs, namely: (i) sociodemographic and professional characterisation, (ii) professional health and occupational risk, (iii) engineering controls and use of PPE, (iv) preparation techniques and quality assurance practices, (v) administrative and organisational safety measures, and (vi) environmental contamination monitoring and control. It consisted predominantly of closed-ended questions with predefined response options to support efficient quantitative analysis. A final open-ended question was included to allow participants to freely express their main concerns and perceived difficulties related to cytotoxic drug handling; however, data were not analysed in the present article. Data were collected over a six-week period, from 14 April to 26 May 2025, through email and professional social media networks to optimize participation among eligible participants. Completion of the questionnaire was conducted through the Office^®^ Forms platform (forms.office.com).

### 2.5. Data Analysis

Descriptive statistics were used to characterise the study population and summarise responses across the different domains. Microsoft Office Excel (version 2603) was used for this purpose. Results were analysed to identify patterns in safety practices, perceived risks, and institutional conditions related to cytotoxic drug handling. Inferential statistical analysis was performed using IBM SPSS Statistics for Windows, version 27.0. Associations between categorical variables were examined using Pearson’s chi-square test; when the assumptions for the chi-square test were not met, exact tests were used, namely Fisher’s exact test for 2 × 2 tables and the Fisher–Freeman–Halton exact test for larger contingency tables. For associations involving ordinal variables, Spearman’s rank correlation coefficient was calculated to assess the direction and strength of monotonic relationships. All tests were two-tailed, and a *p*-value < 0.05 was considered statistically significant.

### 2.6. Ethical Considerations

The study protocol was approved by Ethics Committee of the School of Health of Polytechnic Institute of Porto (CE00036F) on 21 March 2025. Participation was voluntary and anonymous, and informed consent was obtained electronically prior to questionnaire completion. The study complied with the ethical standards of the Declaration of Helsinki [31] and its subsequent amendments.

## 3. Results

A total of 215 completed questionnaires were received. Ninety-one responses were excluded because the professionals were not actively handling cytotoxic drugs at the time of the survey. The final sample was composed of 124 respondents.

### 3.1. Workload and Exposure Patterns

Approximately 50% of respondents had limited experience (less than 3 years), with one-third having less than one year of experience (Table 1). Of the total analysed sample (*n* = 124), 78% (*n* = 97) were female, with ages ranging from 23 to 58 years. Among male professionals, (*n* = 27; 22%) ages ranged from 23 to 60 years.

**Figure 1:**
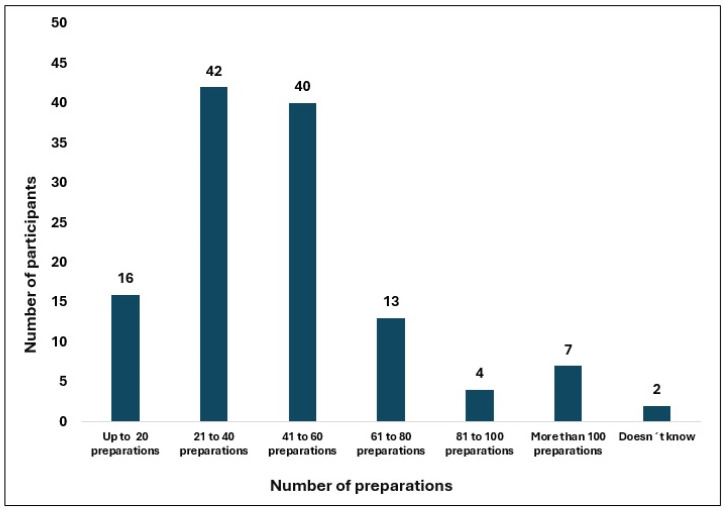
Number of preparations per working day.

**Figure 2:**
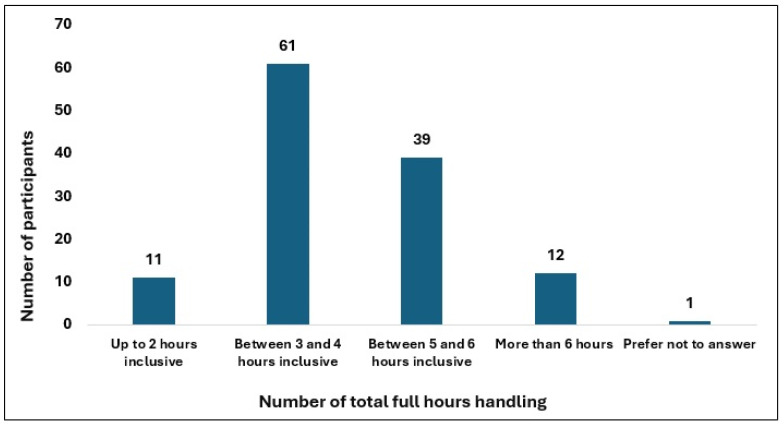
Participant distribution by daily cytotoxic handling hours.

**Figure 3:**
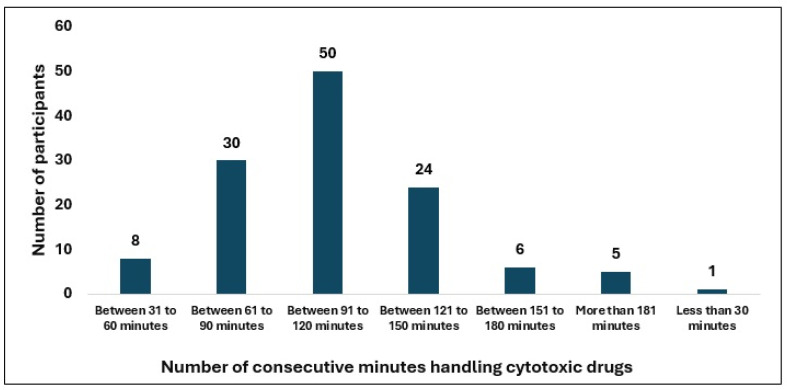
Uninterrupted handling duration.

Most professionals (>80%) reported that their competencies were not assessed prior to starting cytotoxic drug handling, and 71% indicated that no specific training was provided afterward. Approximately 49% of respondents spent 3–4 h per day handling cytotoxic drugs followed by 31% who handled cytotoxic drugs for 5–6 h per day. Regarding the number of preparations performed, most participants fell within 21–40 preparations/day (34%) and 41–60 preparations/day (32%) ranges. Nineteen percent reported performing more than 60 preparations per day, including 5.7% who reported more than 100 preparations daily. The most frequently reported uninterrupted handling durations were 91–120 min (40%) and 28.2% of the participants exceeded 120 min of consecutive handling of cytotoxics—Table 2, Figure 1, Figure 2 and Figure 3.

### 3.2. Administrative and Organizational Safety Measures

Professionals were asked which conditions justified the temporary or permanent suspension from handling cytotoxic drugs. Pregnancy (96.0%), breastfeeding (95.2%), and intention to conceive (91.9%) were the most frequently selected conditions for temporary or permanent suspension from handling cytotoxic drugs (Table 3). In contrast, marked decrease in visual acuity (14.5%) and history of miscarriages or congenital malformations (19.4%) were selected less frequently (see Table 3).

Regarding family planning, most women are removed from cytotoxic drug handling 4–6 months before planned conception (44.4%), while a small proportion continue handling cytotoxic drugs until pregnancy occurs (5.7%). For men, practices are less consistent: the most common approach is removal 4–6 months before conception (23.4%), but 16.1% continue handling cytotoxic drugs until conception. Approximately 16% of participants reported that men were never removed from cytotoxic preparation unit (CPU) duties at their workplace, even when planning to conceive. A high proportion of respondents reported not knowing the policy, particularly for men (30.7%), indicating limited clarity on institutional practices (Table 4).

When asked about washout periods as a preventive measure against musculoskeletal injuries and/or health complications related to cytotoxic drugs handling, more than 50% of the professionals reported that no prophylactic withdrawal period was implemented at their workplace.

Table 5 shows the type of training received prior to starting duties in the CPU, with professionals frequently obtaining their initial training during a Pharmacy degree (*n* = 90, 72.6%). Approximately 62% also report having been trained by experienced peers at their institutions, while 34.7% (*n* = 43) reported that they received structured and formalized training provided by pharmacy service peers. Postgraduate training (postgraduate diploma, master’s, or doctoral degree) was infrequent (3.2%). Participation in short courses offered by educational institutions outside degree programs was reported by 8.1%, while short courses promoted by non-academic entities, such as the pharmaceutical industry, were reported by 5.6%. Notably, 1.6% of respondents reported receiving no training in this area before start of the activity. Despite the bibliographic resources available for consultation during preparation activities, approximately one third of professionals reported that Internal Procedures are consulted to support routine production.

Training records (46.7%) and evaluation/assessment records (17.7%) were observed in a low number of respondents. As to specific content of training, the professionals indicated the need for programs and orientations focusing mainly on the use of PPE, hand hygiene, aseptic technique during handling, existing rules, regulations and recommendations on the handling of cytotoxic drugs, and on spill management.

Regarding professional integration to start activity in CPU, the results show low assessment of competency levels before starting chemotherapy handling. Only 12.9% reported evaluation of their ability to prepare sterile solutions prior to initiating duties. Periodic evaluation was reported as “never” by 68.5% for sterile preparations and by 84.7% for simulated innocuous preparations (Table 6). Professionals were also asked about periodic specialized training in cytotoxic drug handling after starting practice and the frequency of this training. More than 70% reported not receiving any follow-up training since beginning these activities.

### 3.3. Engineering Controls and Technical Resources

Table 7 presents the material resources and engineering controls reported as being available to use for cytotoxic drugs handling. Differences between materials, Luer-lock syringes (94.3%) and pressure-release spikes (94.3%) were widely reported as available. Closed system transfer devices (CSTD) were reported by 60.5% of respondents.

Regarding automated equipment, more than half of the participants (54.0%) reported not using any automated equipment. Equipment was used for agitation or homogenization of solutions (29.8%), filling infusion pumps (24.2%), filling infusion bags (6.5%), and syringe filling (0.8%). Only 2.4% reported using robots to completely replace human operators in cytotoxic drug preparation.

Concerning the type of biological safety cabinet, most participants (61.3%) reported working with Class II B2 cabinets. Other cabinet types were reported less frequently: Class II A2 (5.6%), Class II B3 (4.0%), Class II B1 (2.4%), Class II A1 (1.6%), and Class I and Class III (0.8% each). A total of 26.6% reported not knowing the type of biological safety cabinet available in their unit. Still, in most cases (*n* = 66; 53%), two professionals are present in the cleanroom, with the main duties of the professional assisting the handler being the transfer and verification of materials (such as syringes, needles, infusion pumps, spikes, etc.), drugs, reconstitution solvents, and dilution solutions into the laminar airflow cabinet.

The professionals in the cleanroom are also responsible for verifying labels to ensure that the patient’s name, drug, diluent solution, and volumes correspond to the production sheet; transferring finished preparations to the outside of the cleanroom (83%); and, performing double-checks of measured volumes and double verification of the visible characteristics of the final preparations (such as the formation of precipitates, presence of foreign particles, or abnormal color changes) (64%).

With respect to the spill kit available in the CPU nearly all professionals reported that a kit is present. Most respondents (*n* = 95, 76.5%) believe that its contents are either fully or partially adequate, but 15 respondents reported knowing what the kit contains but are unsure whether it is adequate, 12 respondents do not know the contents of the spill kit at all and 40% of professionals (*n* = 50) state they have never received training on the spill kit (see Table 8).

Regarding aseptic technique validation, 63% of professionals have individual validation of their aseptic technique according to an internal protocol, while only a small number report validation based on national (*n* = 4) or international (*n* = 3) protocols. However, one third of the professionals do not have their aseptic technique validated and 12% are unaware of the existence of aseptic technique validation.

Regarding microbiological monitoring in cleanrooms, although the majority stated that monitoring is performed regularly, approximately 15% (*n* = 19) reported that no routine microbiological monitoring is conducted. It is also noteworthy that 5.7% were unaware of whether such monitoring is performed.

Regarding particle counting, 39 respondents reported not knowing whether particle counting was conducted, and 35 reported that no routine particle counting was performed.

### 3.4. Personal Protective Equipment

Table 9 shows PPE used cross-tabulated with professional experience in handling cytotoxic drugs. High adherence to protective measures was found, with the use of shoe covers reported as a single pair (66.9%) or double pair cover (23.4%). Non-use was reported by 9.7% of professionals across distinct experience levels; still, seven of the twelve professionals who reported not using shoe covers had less than one year of professional experience.

Regarding the gown, all participants reported their use, with most handlers reporting the use of the reinforced version (91.1%), regardless of professional experience. The use of a non-reinforced gown was reported by 8.9% of the participants, with a higher frequency among professionals with more than 10 years of experience. Approximately 70% of participants reported changing gowns upon each entry into the cleanroom. However, 13% stated that they reused the same gown throughout the entire day, across multiple cleanroom entries, even during handling periods exceeding three hours. A total of 84.7% respondents reported using a single hair covering, while 11.3% used two hair coverings. Non-use of hair protection was reported by 4% of participants, mainly among less experienced professionals.

Regarding respiratory protection, FFP3 masks were the most frequently used (56.5%), followed by FFP2 masks (36.3%). Interchangeable use of FFP2 and FFP3 was reported by 7.3% of participants. Variability in mask choice was observed across experience levels, without a clear dominance of a specific group.

Regarding protective goggles, only 30% of professionals reported using them routinely, with higher rates among those with less than one year of experience and those with 7–9 years of experience.

Only 14% of professionals replaced gloves within 30 min of handling. It is important to note that 8% of handlers still use the same pair of gloves throughout the entire consecutive handling period. Double-glove use was reported by 91.9% of respondents (*n* = 114). Single-glove use was reported by 6.5%, and non-use by 1.6%, was limited to two professionals with less than one year of experience. When questioned about which pair of gloves is replaced during the same handling period, the majority (around 80% of handlers) reported replacing only the outer pair, while maintaining the inner pair (in direct contact with the hands) throughout the handling session. According to 66% of the professionals (*n* = 82), the gloves used in the cytotoxic drug preparation unit where they work are clearly labelled as suitable for handling cytotoxic drugs.

### 3.5. Exploratory Inferential Analysis

Exploratory inferential analyses were conducted to assess potential associations between demographic characteristics (sex and age), professional experience, workload patterns, training, and safety practices.

No statistically significant association was observed between professional experience and PPE full compliance (Fisher–Freeman–Halton exact test, *p* = 0.092; *n* = 124). Similarly, no significant association was found between sex and PPE full compliance (Fisher’s exact test, two-sided *p* = 0.460; *n* = 124), nor was age significantly associated with PPE full compliance (Mann–Whitney U test, *p* = 0.058; *n* = 124).

With regard to workload, no statistically significant monotonic association was identified between daily hours handling cytotoxic drugs and competency assessment following initial training (Spearman’s rho = 0.091, *p* = 0.325, *n* = 120). Likewise, no statistically significant association was found between daily handling time and habitual break duration (Spearman’s rho = 0.151, *p* = 0.097, *n* = 121), although a weak positive trend was observed.

The analysis of training and safety infrastructure showed no statistically significant association between the existence of formal training records and the implementation of environmental monitoring (Fisher–Freeman–Halton exact test, *p* = 0.228; *n* = 99).

Sex-based analyses revealed no consistent associations across most variables, including consecutive time working in cytotoxic preparation units (Fisher–Freeman–Halton exact test, *p* = 0.386; *n* = 124), break duration (Fisher–Freeman–Halton exact test, *p* = 0.503; *n* = 122), training records (Fisher’s exact test, two-sided *p* = 1.000; *n* = 108), and competency assessment (Fisher’s exact test, two-sided *p* = 1.000; *n* = 121). Although statistically significant associations were observed between sex and daily handling hours (*p* = 0.020; *n* = 123) and between sex and frequency of microbiological monitoring (*p* = 0.032; *n* = 111), these associations were not supported by significant monotonic correlations (daily handling hours: Spearman’s rho = 0.051, *p* = 0.581; microbiological monitoring frequency: Spearman’s rho = −0.030, *p* = 0.755), suggesting that these findings do not reflect meaningful trends.

Regarding age, a statistically significant but weak positive correlation was identified between age and consecutive time working in cytotoxic preparation units (Spearman’s rho = 0.284, *p* = 0.002; *n* = 122), indicating that older professionals tend to have longer continuous experience in these settings. No other significant associations were observed between age and the remaining variables.

No statistically significant association was observed between the adoption of automation techniques during cytotoxic drug handling and the duration of consecutive handling periods (Fisher–Freeman–Halton exact test, *p* = 0.278; *n* = 124).

Overall, the inferential analyses did not identify strong or consistent associations between demographic or professional variables and safety practices, workload, or training-related factors (Table 10).

### 3.6. Thematic Analysis of Open-Ended Responses

An exploratory thematic analysis was conducted on the responses to the open-ended question regarding professionals’ concerns. Twenty-three participants responded to the open-ended question, presenting a total of 33 concerns that were grouped into eight key themes (Table 11).

In the domain of occupational safety and working conditions, participants reported concerns related to physical symptoms associated with prolonged stays in preparation rooms, as well as issues related to the instability of pressure control systems. At the organizational and human resources level, considerable heterogeneity was observed, including differences in task distribution, lack of support staff, and absence of double-checking procedures in some settings.

Concerns regarding PPE included both inconsistent use and perceived inadequacy in certain contexts. In terms of standardization, several participants highlighted the need for greater uniformity of procedures at the national level, suggesting the development of formal guidelines and the creation of specialized centers for cytotoxic drug handling.

Within the domain of occupational health and medical surveillance, participants reported insufficient medical follow-up and difficulties in implementing preventive measures, such as “wash-out” periods. Finally, issues related to training and knowledge were also identified, including limited access to training and variability in technical practices. Some respondents also provided feedback on the questionnaire, generally expressing a positive perception of its relevance.

## 4. Discussion

This study allowed for a detailed characterization of occupational exposure patterns and the risk perception of professionals involved in handling cytotoxic drugs in Portuguese hospital units, simultaneously revealing heterogeneity on the application of established guidelines. Cytotoxic drug handling practices in Portugal had previously been described in another observational study [32]. That study suggested shortcomings in adherence to recommended practices, particularly regarding staff training and the use of engineering controls [33]. However, it reflected the practices of individuals who had handled cytotoxic drugs at any time between 2017 and 2022, meaning that some participants were no longer actively performing these activities. The present study provides an updated characterization based exclusively on professionals currently handling cytotoxic drugs and includes 61% more active participants than the previous publication. In addition, the most recent ISOPP guidelines were updated in 2022 [1] and may not have been implemented at the time of the earlier survey. Given European regulatory expectations for compliance with ISOPP standards [19,27], it is reasonable to assume that alignment with these strengthened guidelines would be more likely by 2025 [1].

A first important finding, regarding sociodemographic characterization, is the clear trend to female participation, which is consistent with both international and national literature on workers exposed to cytotoxic agents [2,20,32,34,35,36,37,38,39], where the proportion of female professionals typically ranges between 75 and 90 age distribution, our sample shows that most participants are between 31 and 40 years old, again aligning with international findings even in different professional groups similarly exposed to cytotoxic risks, as nurses and pharmacists [2,27,32,34,36,37,38,39,40,41,42]. Accordingly, the sample are young (the majority being under 50 years old) and predominantly women of reproductive age who may become pregnant or breastfeed. Temporary absences of staff members, for instance for family planning purposes, further exacerbate this pressure and help to explain. In fact, this workforce turnover may contribute to a higher proportion of less experienced professionals being involved in the handling of cytotoxic drugs [40]. Approximately one-third of the surveyed have less than one year of experience. This highlights the importance of adequate training, competency assessment prior to assuming duties, and structured onboarding processes. Without these, newly hired staff may not be sufficiently prepared, leading to a significantly higher risk of exposure for themselves and their colleagues, an exposure that is cumulative over time.

### 4.1. Workload and Exposure Patterns

Prolonged (>120 min), uninterrupted work periods inside the biological safety cabinet were observed in almost 30% of the participants, and team sizes may be insufficient to prevent cumulative exposure. Such circumstances raise questions about the extent to which current practices comply with established guidelines [1,3]. Although none of the major international guidelines define a strict maximum number of hours per day for cytotoxic drug handling, all emphasize the need to minimize cumulative exposure and to avoid prolonged continuous manipulation. The ISOPP’s, ASHP and USP <800> guidelines recommends limiting uninterrupted work inside biological safety cabinets and introducing regular rest breaks, typically 15 min after every 60 to 90 min of continuous handling, to mitigate fatigue, muscle tremor, ergonomic strain, operational errors and contamination risk [1,3,16].

In the present study, more than 40% of respondents reported compounding cytotoxic drugs for 91–120 min without interruption, and approximately 20% exceeded 120 min. These durations may increase the potential for compromised aseptic technique, environmental contamination, and inadvertent exposure [1,3,7,16]. An important proportion of participants reported no breaks at all, further underscores the need to review organizational models and human-resource allocation. Consistent with these observations, the total daily workload in some cases might also exceed safe ergonomic and operational standpoint, given the prolonged use of PPE, fixed postures, and repetitive tasks. In this study, approximately two-thirds of professionals reported compounding 21–60 preparations per day, but some respondents admitted exceeding 100 preparations/day. However, these numbers also depend on the type of preparation. Such high activity levels may compromise safety may increase the potential for exposure, operational errors, fatigue and muscle problems if not supported by adequate staffing, task rotation, and systematic fatigue monitoring [33]. Although no guideline establishes a maximum number of daily preparations, it is commonly emphasized that the workload must remain compatible with maintaining technical accuracy and strict adherence to individual and collective protective measures.

With respect to total hours of daily exposure, nearly 90% of the participants reported handling cytotoxic drugs for more than three hours per day. International guidelines recommend organizational measures aimed at reducing cumulative exposure, including limiting continuous handling time and distributing preparation activities among staff, which in practice implies systematic task rotation [1,3,7,16,33]. Once again, the findings of this study indicate that rotation practices are not implemented uniformly at the national level, highlighting a potential area for targeted intervention. Poor workflow design and staff shortages are primary contributors to physical and cognitive overload and may further compromise the ability of services to respond to the increasing workload, while also contributing to increased absenteeism and staff leave [7,43].

The assessment of clinical conditions warranting temporary or permanent exclusion from cytotoxic drug compounding revealed marked discrepancies across distinct risk categories. There was remarkably high consensus (>90%) regarding the need to restrict compounding activities for pregnant or breastfeeding workers, as well as those planning to conceive. This strong agreement suggests an important level of institutional awareness of reproductive risks associated with cytotoxic exposure, which are traditionally well-recognized and thoroughly addressed during initial professional training [1,3,7,17,18]. Occupational exposure to cytotoxic drugs has been associated with an increased risk of infertility, genetic damage (mutations), spontaneous abortions, fetal malformations, and impaired embryonic development [5,44,45,46]. In contrast, substantially lower agreement was observed for several conditions that are referenced in international safety guidelines for the handling of hazardous drugs, typically as context-dependent indicators of fitness for duty rather than explicit exclusion criteria, including acute respiratory or gastrointestinal illnesses, recent hematological abnormalities, reduced visual acuity, and certain chronic diseases [1,3,7]. The low risk perception associated with visual acuity, identified in this study, constitutes a relevant and potentially concerning finding. In fact, visual acuity plays a fundamental role in the handling of cytotoxic drugs, being essential for the visual detection of precipitates, foreign particles, and color changes, as well as for the accurate verification of volumes during preparation. These steps are critical and essential to ensure the quality and safety of preparations, having a direct impact on patient safety. According to international recommendations, namely from ISOPP [1], visual inspection constitutes a mandatory step in the process of preparing cytotoxic drugs and should be carried out under appropriate conditions and by professionals with adequate visual capacity. The underestimation of this factor can compromise the detection of non-conformities, increasing the risk of administering defective preparations, with potential clinical consequences. In this context, it becomes relevant to reinforce the importance of periodic assessment of the visual acuity of professionals involved in handling, as well as the implementation of adequate lighting conditions and strategies that minimize visual fatigue. The integration of these aspects into safety and training programs could contribute to an overall improvement in the quality of practices and patient safety. Accordingly, risk perception may be incomplete in areas less emphasized in training programmes or internal protocols, despite evidence that such conditions may impair adherence to protective equipment, reduce technical precision, or limit the ability to respond effectively to spill incidents. The results indicate that existing institutional policies are more formalized, and considerably more conservative, for women, particularly regarding preconception and pregnancy, reflecting a well-established recognition that occupational exposure to cytotoxic drugs can affect fertility and early embryonic development [3,44,45,46,47]. However, these policies appear to place less emphasis on male reproductive risks, despite evidence that cytotoxic agents may induce oligospermia, sperm DNA damage, and broader adverse effects on male fertility [16]. This difference may partly reflect the shorter window of perceived risk for women, who have a monthly ovulatory cycle, compared with men, whose spermatogenesis cycle takes approximately 60 days. Consequently, male reproductive risks may be underestimated in institutional policies, even though exposure during the spermatogenic period could significantly affect fertility and embryo health. This gap is reflected in the substantially lower agreement across all categories concerning the removal of men from compounding duties, the markedly higher proportion of “I don’t know” responses, and the finding that 16% of participants work in institutions that never withdraw male workers from chemotherapy handling, even during family planning. On the other hand, most participants work in hospital units that protect women from cytotoxic handling several months before conception, typically 4 to 6 months. Nonetheless, a minority of institutions allow women to continue compounding until pregnancy is confirmed, a practice that runs counter to precautionary approaches to reproductive protection [7,17,18,23,27].

This asymmetry warrants further consideration in light of how reproductive risk has been historically framed in occupational health systems. In many healthcare settings, preventive policies concerning hazardous drug exposure have been shaped primarily around pregnancy protection, making female reproductive risk more visible, more formalised, and more readily translated into workplace procedures. By contrast, male preconception exposure has often received less explicit institutional attention, not because the risk is necessarily absent, but paternal reproductive risk has been less clearly incorporated into routine occupational guidance, surveillance, and risk-management protocols. This interpretation is especially relevant in the context of cytotoxic drug handling. Reproductive risk from hazardous medicines is not exclusively female, and paternal exposure in the preconception period should not be assumed to be negligible. Yet legal frameworks, occupational-health practice, and institutional policies have not always evolved symmetrically. Consequently, women are more often covered by specific protective pathways linked to pregnancy and maternity, whereas men may remain subject to broader, less explicit, and less operationalised forms of risk management. This imbalance may help explain why workplace practices appear comparatively more standardised for women and substantially more ambiguous for men. In settings where hazardous drugs are handled routinely, the absence of clear preconception guidance for both sexes may create avoidable occupational-health, ethical, and medico-legal vulnerability for institutions.

Overall, the findings reveal a substantial lack of standardization and highlight the urgent need for clear national protocols grounded in international occupational safety guidelines to ensure consistent and evidence-based protection for both female and male workers [48,49,50].

The exploratory inferential analyses did not reveal strong or consistent associations between demographic characteristics, professional experience, and key indicators of safety practices, workload, or training. These findings suggest that variability in occupational practices may be more strongly influenced by structural and organizational factors than by individual characteristics alone. Although some statistically significant associations were identified, their lack of consistent directional trends and weak correlation coefficients indicate limited practical relevance. This reinforces the importance of system-level approaches when addressing occupational safety in cytotoxic drug preparation settings.

The workload patterns identified in this study suggest sustained exposure to cytotoxic drug handling, characterized by prolonged daily activity, extended uninterrupted handling periods, and high preparation volumes. These factors are recognized in occupational settings as contributors to cumulative exposure and task-related risk. However, the interpretation of exposure intensity in this context remains complex, as it is influenced not only by duration and frequency but also by factors such as preparation complexity, drug characteristics, and technical conditions. The absence of well-established, context-specific exposure thresholds further highlights the need for cautious interpretation. Nevertheless, the observed patterns provide important insight into working conditions that may be associated with increased occupational risk.

### 4.2. Administrative and Organizational Safety Measures

The findings of the aseptic technique assessment reveal a substantial heterogeneity, even though such competencies should be formally evaluated prior to independent practice and revalidated at regular intervals, typically annually in accordance with international guidelines [1,3,7,16]. The high proportion of professionals who reported not undergoing any validation or who are unaware of its existence suggests the absence of structured competency-certification programs; deficiencies in internal communication and documentation systems; inconsistencies in training pathways; and an overall lack of harmonised quality-assurance procedures. Collectively, these shortcomings may translate into an increased risk of aseptic breaches, medication preparation errors, and heightened occupational exposure issues consistently highlighted as preventable by NIOSH, ISOPP, ASHP, and EAHP [1,7,15,16,17,18]. More than 70% of participants reported not having received any specific follow-up training since beginning their handling activities, which highlights a substantial gap in ongoing competency reinforcement, despite the recognised need for continuous education in high-risk pharmaceutical preparation environments [1,3,7,16,51].

Although most respondents reported that regular microbiological monitoring is performed, 15% of participants work in units where no routine microbiological surveillance is conducted. The wide variability in monitoring frequency (ranging from daily to monthly) further underscores the lack of national standardization [1,3,7,15,16], deficiencies in communication, transparency, and staff engagement with quality-assurance processes. The absence of routine microbiological monitoring, as well as the reported lack of knowledge regarding particle counting, constitute concerning findings. In Portugal, although there is no specific legislation exclusively dedicated to handling cytotoxic drugs that exhaustively details all environmental monitoring requirements, these practices are framed within broader standards related to the preparation of sterile drugs and cleanroom control. In this context, ISOPP [1] recommendations, as well as references such as USP <800> [3], advocate the implementation of environmental monitoring programs, including microbiological control and particle counting, as essential components to ensure the quality and safety of sterile preparations. Thus, the observed variability may reflect not only differences in the interpretation and implementation of these recommendations, but also the absence of specific national standards applied uniformly, with many of these practices depending on the internal policies of the institutions. This fact reinforces the need for greater harmonization and regulatory clarification, to ensure consistent levels of quality and safety across all institutions. Cleanrooms without microbiological monitoring may represent a substantial risk to the sterility of compounded preparations, a concern that is particularly critical during the reconstitution of cytotoxic drugs [52]. Evidence shows that even when isolators and biological safety cabinets are used, contamination of work surfaces—and occasionally of intermediate products—with antineoplastic agents can still occur, underscoring that engineering controls alone do not prevent contamination without consistent environmental and process monitoring [53,54,55,56,57]. Comparative studies show that units implementing formalized aseptic procedures, staff training, and systematic monitoring achieve significantly lower contamination levels [52,57,58,59,60].

The results also reveal elevated level of non-compliance in particle monitoring compared with international benchmarks [1,3,7,16] According to ISOPP and ASHP recommendations, at minimum, annual (preferably semi-annual) particle monitoring, depending on risk assessment and classification of the compounding environment [1,16]. Continuous or frequent monitoring in critical areas and periodic monitoring in support zones are also recommended [1,16]. The high number of professionals who report not knowing whether particle monitoring occurs (*n* = 39) or state that no routine monitoring is performed (*n* = 35), suggests an absence of robust environmental qualification processes and a lack of professional integration into quality activities. The pattern further suggests a potential over-reliance on PPE as a compensatory measure, rather than ensuring adequate performance of primary and secondary environmental controls. Despite the importance of PPE, it should be emphasized that, within the hierarchy of controls for preventing occupational exposure, PPE is considered the least effective measure [20]. In contrast, engineering controls—including cleanrooms, biological safety cabinets, and their operating conditions—are regarded as the most effective means of exposure control when elimination or substitution of these drugs is not feasible [1].

Taken together, the combined analysis of aseptic technique validation, microbiological monitoring, and particle monitoring indicates marked heterogeneity and incomplete adherence to international guidelines [16,29,47]. This fragmented landscape highlights the urgent need for national harmonization of procedures, strengthening of environmental monitoring programs, and improved communication strategies to ensure that all staff are informed, trained, and actively involved in quality and safety systems.

Training is primarily acquired during undergraduate education and/or through informal workplace-based learning, such as peer mentoring. These findings support the need for more structured, documented, and standardized training programs.

The findings reveal a substantial deficiency in structured training at both the initial and periodic levels for cytotoxic drug compounding. The results shown, in Table 5, point to the absence of formal certification and recertification programmes, in sharp contrast with international guidelines [1,3,7,16,18] and recent systematic reviews [51], which emphasize mandatory pre-placement evaluation and regular reassessment as essential pillars of safety in cytotoxic compounding.

Moreover, the limited use of innocuous-solution testing represents a missed opportunity for safe practice, hands-on skills reinforcement, and early detection of technical errors, all of which contribute to sustained process reliability and reduced occupational risk.

Globally, the findings suggest that safety in cytotoxic drug handling is primarily supported by locally acquired practices and experiential learning, rather than by fully structured, auditable, and standardized systems. Strengthening formal continuing education, documentation, competency monitoring, and integration of international guidelines could enhance practice harmonization and further reduce occupational risk.

### 4.3. Engineering Controls and Technical Resources

Most professionals reported that standard preparation materials—including needles, luer-lock^®^ syringes and spikes—are available and routinely used. It is worth highlighting that luer-lock^®^ connections should be preferred over slip-tip connections due to their greater stability when attached to the puncture device, thereby reducing the likelihood of detachment [2,61]. This study illustrates clear changes in Portuguese practice over a relatively short period, as adherence to CSTD use has increased compared with results published by Campos et al. [32], in which CSTD use was virtually non-existent. Nevertheless, given the substantial proportion of professionals who still do not have access to or do not use these devices, it will be important to reinforce recommendations for their use, as they offer greater safety compared to other materials [62,63]. In line with observations of inconsistent adoption of CSTDs, a survey of UK cancer nurses also identified variable use of closed handling systems and protective measures, highlighting that even in resource-rich settings, the adoption of specific safety practices remains inconsistent [64,65]. Variability in the use of CSTDs and other advanced controls has been reported in other settings, indicating that such gaps are not unique to the Portuguese context [32,66].

Only 30% to 46% of respondents report having access to automated equipment, such as devices for bag filling, agitation/homogenization, or pump filling. Their use could prevent musculoskeletal injuries, particularly affecting the upper limbs, including the shoulders, wrists, and fingers [1,19]. Moreover, despite the costs associated with acquisition, implementation, and maintenance, automated systems may be regarded as valuable solutions for preserving human resources [45]. Particularly regarding the very low adoption of automated or robotic systems observed in this study (2.4%), it most likely reflects substantial economic and infrastructural barriers, including high initial investment and implementation costs. However, these technologies may offer important long-term benefits besides the prevention of musculoskeletal injuries already mentioned, such as reduced occupational exposure, fewer preparation errors, and improved process consistency. From a broader perspective, these gains may translate into indirect cost savings through the prevention of occupational disease and adverse events, supporting the consideration of automation as a potentially cost-effective strategy for improving safety and quality in cytotoxic drug handling.

### 4.4. Personal Protective Equipment

In the present study, adherence to the use of mandatory PPE was very high and, overall, higher than that reported in most comparable studies published since 2004, the year in which NIOSH formally introduced the definition of hazardous drugs and subsequently shaped occupational safety recommendations [2,9,32,41,67,68,69,70,71]. Glove use was almost universal, and our findings stand out due to near-complete compliance with protective gowns. Adherence to respiratory protection and head cover was also notably higher than in many earlier studies, where these items often showed considerable variability or substantially lower uptake [2,9,69,70,72]. In contrast, the use of overshoes was more inconsistent. From an aseptic workflow perspective, the use of a single pair of shoe covers does not ensure adequate environmental protection during transitions between areas of different cleanliness levels. According to the ISOPP Standards of Practice, PPE must function as an effective barrier system to prevent cross-contamination between controlled and adjacent areas. In practical terms, this requires the use of double shoe covers: one pair worn upon entry into the grey area and a second pair added before entering the cleanroom, allowing the outer pair to be removed upon exit from the cleanroom while maintaining environmental protection in the grey area [1]. When this functional definition of compliance is applied, more than three-quarters of respondents in our study may be considered non-compliant with best practices for foot protection.

Overall, this study highlights a dual reality: while compliance with long-established protective measures is generally high, several critical components of occupational safety—particularly workload management, environmental monitoring, and structured training—remain inconsistently implemented. This inequality is a systemic, not an individual, risk-governance issue, highly relevant in the European and CMR contexts.

### 4.5. Thematic Analysis of Open-Ended Responses

The qualitative findings obtained through the thematic analysis of open-ended responses reinforce and contextualize the quantitative results, providing additional insight into the challenges associated with cytotoxic drug handling. Concerns related to occupational safety and environmental conditions—particularly physical symptoms associated with prolonged stays in preparation rooms and instability of pressure differentials—are consistent with the literature, which identifies environmental control as a critical factor in minimizing occupational exposure [43].

The heterogeneity observed in work organization, including the absence of double-checking procedures and support staff in some settings, raises important concerns regarding process safety. International recommendations, such as those from ISOPP [1], emphasize that cytotoxic drug handling should occur in controlled environments with standardized procedures, including measures to reduce both exposure and error risk.

The need for greater standardization, frequently highlighted by participants, points to a gap at the national level. The absence of consistent guidelines may contribute to variability in practices and inequalities in professional protection. This is particularly relevant given that international organizations recommend clear standards for PPE use, work organization, and environmental monitoring.

Additionally, concerns related to occupational health surveillance—namely insufficient medical follow-up and difficulties in implementing preventive measures such as “wash-out” periods—suggest gaps in current protective frameworks. These findings align with the broader results of this study, reinforcing the perception that occupational risks associated with cytotoxic drug exposure may be insufficiently addressed.

Overall, the integration of these qualitative data provides a more comprehensive understanding of the realities experienced by professionals, highlighting not only technical issues but also organizational and human factors that should be considered when developing strategies to improve safety in cytotoxic drug handling.

### 4.6. Strengths and Limitations

This study has several important strengths. First, it provides a comprehensive and up-to-date characterization of cytotoxic drug handling practices among pharmacy technicians in Portugal, focusing exclusively on professionals who were actively involved in cytotoxic preparation at the time of data collection. This represents a significant improvement over previous national studies, which included only professionals with prior exposure and had smaller sample sizes. The relatively large sample size (*n* = 124) enhances the descriptive power of the findings and enables a detailed analysis of training pathways, workload patterns, engineering controls, PPE use, and administrative safety measures. Second, the questionnaire was developed through a rigorous, multi-stage process grounded in international guidelines and expert consensus, including a focus group of experienced professionals and a pre-test with the target population. This approach enhanced the content validity and practical relevance of the instrument. Although the internal consistency was moderate (Cronbach’s α = 0.648), this value is considered acceptable for an exploratory study covering multiple distinct domains of occupational safety, rather than a single latent construct. The inclusion of both technical and organizational dimensions provides a more comprehensive perspective on occupational exposure risk that extends beyond PPE compliance alone. Third, the direct comparison of current practices with international reference standards (ISOPP, ASHP, NIOSH, USP <800>) represents a key strength, as it enables the identification of concrete gaps, intervention priorities, and opportunities for harmonization at the institutional and national levels. The study also highlights underexplored areas, such as workload management, consecutive handling time, and the recognition of non-reproductive clinical conditions that may contraindicate cytotoxic handling.

However, several limitations must be acknowledged. Data were collected through a self-administered questionnaire, which may be subject to recall bias and social desirability bias, potentially leading to overestimation of adherence to recommended practices. Moreover, responses were collected at the individual level, and multiple participants may have originated from the same hospital unit. This clustering effect may overrepresent certain institutional practices and limit the generalizability of findings to all Portuguese hospital pharmacy services. While demographic and professional characteristics such as age, sex, and years of experience were collected, information on the workplace was deliberately not obtained to preserve participant anonymity. In smaller centers, the combination of these variables could allow indirect identification of individuals. Consequently, clustering by institution could not be assessed, and the findings should be interpreted as reflecting individual-level practices and perceptions rather than institutional-level prevalence. This may have influenced reported rates of training, environmental monitoring, and availability of engineering controls, which are likely to vary across institutions. Additionally, the cross-sectional design precludes causal inference and does not allow for assessment of temporal changes or the impact of specific interventions. The study did not include direct observational assessments, environmental contamination measurements, or biological monitoring data, which would have strengthened the evaluation of actual exposure levels and compliance. Finally, although the questionnaire covered a wide range of safety dimensions, the moderate internal consistency reflects the heterogeneity of the constructs assessed and suggests that future research could benefit from the development of domain-specific validated subscales.

## 5. Conclusions

This study provides a comprehensive characterization of occupational safety practices in cytotoxic drug preparation units in Portuguese hospitals. While adherence to core protective measures—such as reinforced gowns, double gloving, and Class II B2 biological safety cabinets—appears satisfactory, safety practices remain uneven and structurally fragile. Importantly, the findings suggest that occupational protection is still largely dependent on individual compliance rather than consistently embedded systemic safeguards. Prolonged uninterrupted handling periods, high daily workloads, insufficient rest breaks, limited automation, and incomplete implementation of closed system transfer devices indicate that risk mitigation is not fully supported by organizational design. Training practices further reinforce this structural vulnerability. Although most professionals receive initial academic preparation, structured, continuous, and documented competency assessment is frequently absent, limiting traceability and quality assurance. The high proportion of professionals without regular follow-up training underscores the need for structured, periodic competency assessment frameworks, aligned with internationally recognized safety standards, to ensure consistent practice and reduce occupational risk. This reliance on informal learning and peer mentoring shifts responsibility toward individual professionals rather than institutional systems. The absence of nationally harmonized policies governing cytotoxic drug preparation in Portugal contributes to inter-institutional variability and weakens the standardization of safety practices. Moving forward, improvements should prioritize structural interventions—including workload management, systematic task rotation, strengthened environmental monitoring, mandatory continuous competency assessment, and the development of unified national guidelines aligned with international standards. Strengthening occupational safety in cytotoxic drug handling requires transitioning from a model centered on individual protective behaviour to one grounded in systemic, standardized, and organizationally embedded safeguards.

## Figures and Tables

**Table 1:** Socio-demographic characterization of the participants (*n* = 124).

Variable	Category	Female (*n*)	Male (*n*)	*n* (%)
Total sample		97	27	124 (100%)
Age (years)	≤30	21	8	29 (23.4%)
	31–40	45	13	58 (46.8%)
	41–50	23	5	28 (22.6%)
	51–60	8	1	9 (7.3%)
	>60	0	0	0
Years of experience in cytotoxic drug handling	≤1 years	32	8	40 (32.3%)
	1–3 years	24	3	27 (21.8%)
	4–6 years	13	6	19 (15.3%)
	7–9 years	12	3	15 (12.1%)
	>10 years	16	7	23 (18.5%)

**Table 2:** Cytotoxic drug handling routines vs. years of experience (*n* = 124).

Variable		Experience in Years					
		<1	1–3	4–6	7–9	>10	Total (%)
Daily hours spent handling cytotoxic drugs	≤2	4	5	1	0	1	11 (8.9%)
	3–4	22	11	8	10	10	61 (49.2%)
	5–6	12	8	9	4	6	39 (31.5%)
	>6	2	2	1	1	6	12 (9.7%)
	Prefer not to answer	0	1	0	0	0	1 (0.8%)
Number of preparations performed per day	<20	7	8	0	0	1	16 (12.9%)
	21–40	22	5	4	4	7	42 (33.9%)
	41–60	6	9	11	9	5	40 (32.3%)
	61–80	3	1	3	1	5	13 (10.5%)
	81–100	1	1	0	1	1	4 (3.2%)
	>100	1	2	1	0	3	7 (5.7%)
	Don’t know	0	1	0	0	1	2 (1.6%)
Consecutive handling time without interruption (minutes)	≤30	0	1	0	0	0	1 (0.8%)
	31–60	1	1	0	1	5	8 (6.5%)
	61–90	9	9	3	4	5	30 (24.2%)
	91–120	21	11	9	4	5	50 (40.3%)
	121–150	7	2	7	4	4	24 (19.4%)
	151–180	2	1	0	2	1	6 (4.8%)
	>181	0	2	0	0	3	5 (4.0%)
Common duration of breaks (minutes)	No breaks	4	3	0	2	1	10 (8.1%)
	≤15	5	3	1	1	5	15 (12.1%)
	16–30	12	8	8	5	7	40 (32.3%)
	31–45	9	7	5	3	5	29 (23.4%)
	46–60	6	2	2	0	2	12 (9.7%)
	61–90	3	1	1	1	1	7 (5.7%)
	>90	1	1	2	3	2	9 (7.3%)
	Don’t know	0	2	0	0	0	2 (1.6%)

**Table 3:** Conditions for temporary or permanent suspension from handling cytotoxic handling (*n* = 124).

Conditions	*n* (%)
Pregnancy (current)	119 (96.0%)
Breastfeeding (current)	118 (95.2%)
Family planning (intention to conceive)	114 (91.9%)
History of oncological diseases (past)	89 (71.8%)
Previous treatment with cytotoxic drugs	79 (63.7%)
History of autoimmune diseases (current or past)	54 (43.5%)
Recent changes in routine blood tests	51 (41.1%)
Previous treatment with ionizing radiation	42 (33.9%)
Acute gastrointestinal illnesses (diarrhea, nausea)	41 (33.1%)
Acute respiratory illnesses (at least one of the following symptoms: cough, nasal congestion, cold)	33 (26.7%)
Joint pain	30 (24.2%)
Women with a history of miscarriages and/or congenital malformations	24 (19.4%)
History of anemia (current or past)	23 (18.5%)
Marked decrease in visual acuity	18 (14.5%)
Other	13 (10.5%)
Don’t know	1 (0.8%)

**Table 4:** Time for professional removal from the cytotoxic preparation unit (CPU), assuming planned pregnancy.

Specifically, regarding family planning, in the CPU where you handle cytotoxic drugs, how long before conception are women removed from the CPU (assuming planned pregnancy)?	*n* (%)
Up to 3 months inclusive	10 (8.1%)
Between 4 and 6 months inclusive	55 (44.4%)
Between 7 and 9 months inclusive	13 (10.5%)
Between 10 and 12 months inclusive	10 (8.1%)
More than 12 months	11(8.9%)
None—women continue handling until pregnancy occurs	7 (5.7%)
None—women continue working in the unit until pregnancy, although without handling	1 (0.8%)
Don’t know	16 (12.9%)
Prefer not to answer	1(0.8%)
Specifically, regarding family planning, in the CPU where you handle cytotoxic drugs, how long before conception are men removed from CPU (assuming planned pregnancy)?	*n* (%)
Up to 3 months inclusive	15 (12.1%)
Between 4 and 6 months inclusive	29 (23.4%)
Between 7 and 9 months inclusive	7 (5.7%)
Between 10 and 12 months inclusive	4 (3.2%)
More than 12 months	6 (4.8%)
None—men continue handling until conception	20 (16.1%)
Don’t know	38 (30.7%)
Prefer not to answer	5 (4.0%)

**Table 5:** Administrative Controls.

Type of training specifically related to cytotoxic handling	*n* (%)
Undergraduate training during Bachelor’s/Pharmacy degree	90 (72.6%)
Postgraduate training (Postgraduate, Master’s, or Doctorate degree)	4 (3.2%)
Short courses promoted by educational institutions not included in degree programs	10 (8.1%)
Short courses promoted by non-educational institutions (pharmaceutical industry, medical device labs)	7 (5.6%)
Mentoring and/or shadowing by more experienced pharmacy service peers	77 (62.1%)
Structured and formalized training provided by pharmacy service peers (detailed plan with objectives and record)	43 (34.7%)
Structured and formalized training in the pharmacy service by external entities (detailed plan with objectives and record)	1 (0.8%)
Previous training and/or experience in another hospital unit handling cytotoxic drugs	18 (14.5%)
No training	2 (1.6%)
Bibliographic sources	*n* (%)
Updated list of cytotoxic drugs under preparation	59 (47.6%)
Summary tables of preparation procedures and/or reconstitution solvents and/or dilution solutions	79 (63.7%)
Institutional protocols	41 (33.1%)
Internal procedure manual	71 (57.3%)
Work instructions	58 (46.8%)
Summaries of Product Characteristics (SPCs)	65 (52.4%)
Technical documentation provided by laboratories (not SPCs)	28 (22.6%)
Integration manual for new professionals	22 (17.7%)
International standards	12 (9.7%)
National guidelines	9 (7.3%)
Safety data sheets	25 (2.0%)
Other bibliography	10 (8.1%)
None	1 (0.8%)
Don’t know	5 (4.0%)
Formal Record of Training	*n* (%)
Yes, there are training records	38 (30.6%)
Yes, there are evaluation records	2 (1.6%)
Yes, there are training and evaluation records	20 (16.1%)
No records	48 (38.7%)
Don’t know	15 (12.1%)
Prefer not to answer	1 (0.8%)

**Table 6:** Formal evaluation of compounding abilities.

	Evaluation on the ability to prepare sterile solutions before starting duties	Evaluation on the ability to prepare simulated innocuous solutions before starting duties
Yes	16 (12.9%)	17 (13.7%)
No	104 (83.9%)	103 (83.1%)
Don’t know	3 (2.4%)	3 (2.4%)
Prefer not to answer	1 (0.8%)	1 (0.8%)
	Periodical evaluation on the ability to prepare sterile solutions	Periodical evaluation on the ability to prepare simulated innocuous solutions
Every 3 months (or less)	11 (8.9%)	2 (1.6%)
Between 4 and 6 months inclusive	0	0
Between 7 and 9 months inclusive	0	0
Between 10 and 12 months	5 (4.0%)	3 (2.4%)
Longer than 12 months	6 (4.8%)	7 (5.6%)
Randomly, no defined periodicity	15 (12.1%)	3 (2.4%)
Never	85 (68.5%)	105 (84.7%)
Prefer not to answer	2 (1.6%)	4 (3.2%)

**Table 7:** Material Resources and Engineering Controls available and used for handling cytotoxic drugs.

Material Resources and Engineering Controls		*n* (%)
Materials	Needles	116 (93.6%)
	Luer-lock^®^ syringes	117 (94.3%)
	Pressure release systems, also called spikes	117 (94.3%)
	Closed system transfer devices (CSTD)	75 (60.5%)
	Don’t know/None	0
Automated equipment	For filling infusion pumps	30 (24.2%))
	For agitation/homogenization of solutions	37 (29.8%)
	For filling infusion bags	8 (6.5%
	For filling syringes	1 (0.8%)
	Robots for complete replacement of humans in cytotoxic preparation	3 (2.4%)
	No	67 (54.0%)
	Don’t know	1 (0.8%)
Type of cabinet	Class I	1 (0.8%)
	Class II A1	2 (1.6%)
	Class II A2	7 (5.6%)
	Class II B1	3 (2.4%)
	Class II B2	76 (61.3%)
	Class II B3	5 (4.0%)
	Class III	1 (0.8%)
	Don’t know	33 (26.6%)
	Prefer not to answer	1 (0.8%)

**Table 8:** Spill kit in the cytotoxic preparation unit.

Kit Availability	Category		Formation	
		*n* (%)	Yes (*n*)	No (*n*)
Yes	Fully adequate	54 (43.5)	44	10
	Partially adequate	41 (33.0)	24	17
	Fully inadequate	1 (0.80)	0	1
	I know the content, but I don’t know what the appropriate content of a spill kit should be	15 (12.1)	5	10
	I don’t know the content of the spill kit	12 (9.7)	0	12
	Total	123 (99.2)	73	50

**Table 9:** Protective Equipment available and used for handling cytotoxic drugs in the hospital unit.

Personal Protective Equipment	Variables	<1 Year	1–3 Years	4–6 Years	7–9 Years	>10 Years	*n* (%)
Shoe covers	Not used	7	0	1	1	3	12 (9.7%)
	Single pair	26	19	11	7	20	83 (66.9%)
	Double pair	7	8	7	7	0	29 (23.4%)
Gown	Not used	0	0	0	0	0	0
	Reinforced gown	38	23	18	14	20	113 (91.1%)
	Non-reinforced gown	1	2	1	2	4	10 (8.1%)
	Double gown (non-reinforced)	0	0	0	0	1	1 (0.8%)
Gloves	Not used	2	0	0	0	0	2 (1.6%)
	Single pair	0	2	1	1	4	8 (6.5%)
	Double pair	38	25	18	14	19	114 (91.9%)
Mask	Not used	0	0	0	0	0	0
	FFP2	10	13	4	6	12	45 (36.3%)
	FFP3	26	13	15	8	8	70 (56.5%)
	FFP2 or FFP3	4	1	0	1	3	9 (7.3%)
Hair covering	Not used	2	1	2	0	0	5 (4.0)
	One hair cover	33	23	15	11	23	105 (84.7%)
	Two hair covers	5	3	2	4	0	14 (11.3%)
Protective goggles	Not used	29	19	12	9	17	86 (69.4%)
	Used	11	8	7	6	6	38 (30.6%)

**Table 10:** Summary of exploratory inferential analyses.

Variables Compared	Test Used	Statistic	*p*-Value	*n*	Interpretation
Experience × PPE Full compliance	Fisher–Freeman–Halton	—	0.092	124	Not significant
Sex × PPE Full compliance	Fisher’s exact	—	0.46	124	Not significant
Sex × Daily handling hours	Fisher–Freeman–Halton	—	0.02	123	Significant (no monotonic trend)
Sex × Microbiological monitoring frequency	Fisher–Freeman–Halton	—	0.032	111	Significant (no monotonic trend)
Sex × Break duration	Fisher–Freeman–Halton	—	0.503	122	Not significant
Sex × Consecutive handling time	Fisher–Freeman–Halton	—	0.386	124	Not significant
Automation techniques × Consecutive handling time	Fisher–Freeman–Halton	—	0.278	124	Not significant
Sex × Training records	Fisher’s exact	—	1.00	108	Not significant
Sex × Competency assessment	Fisher’s exact	—	1.00	121	Not significant
Training records × Environmental monitoring	Fisher–Freeman–Halton	—	0.228	99	Not significant
Age × PPE Full compliance	Mann–Whitney U	—	0.058	124	Not significant
Daily hours × Competency assessment	Spearman’s rho	0.091	0.325	120	Not significant
Daily hours × Break duration	Spearman’s rho	0.151	0.097	121	Not significant
Age × Time in unit	Spearman’s rho	0.284	0.002	122	Weak positive correlation

**Table 11:** Summary and grouping of responses provided for open-ended questions.

Theme/Category	Number of Mentions in Open-Ended Question
Occupational safety and working conditions	5
PPE and protection	1
Work organization and human resource	6
Lack of standardization/need for guidelines	5
Occupational health and medical surveillance	3
Training and knowledge (technical practices and variability)	5
Questionnaire feedback (methodological)	5
Overall perception (positive)	3

## Data Availability

The data presented in this study are available on reasonable request from the corresponding author due to confidentiality and privacy restrictions. In accordance with the informed consent provided to participants, the responses are stored securely, accessible only to the research team, and will be deleted five years after publication of the articles resulting from the study, in compliance with applicable data protection legislation.
